# Malignancy of Urinary Tract in Kidney Transplant Recipients—A Narrative Review

**DOI:** 10.3390/cancers18040695

**Published:** 2026-02-20

**Authors:** Sławomir Jerzy Małyszko, Letycja Rog, Ben Sprangers, Amanda DeMauro Renaghan, Mitchell H. Rosner, Rafal Stec, Leszek Kraj, Jacek Stanisław Malyszko, Jolanta Małyszko

**Affiliations:** 1 Department of Physiology and Pathophysiology, Medical University of Warsaw, 02-091 Warsaw, Poland; slawomir.malyszko@wum.edu.pl; 2 Department of Oncology, Medical University of Warsaw, 02-091 Warsaw, Poland; letycja.rog@wum.edu.pl (L.R.); rafal.stec@wum.edu.pl (R.S.); leszek.kraj@wum.edu.pl (L.K.); 3 Department of Nephrology, General Hospital Oost-Limburg, 3600 Genk, Belgium; ben.sprangers@zol.be; 4 Biomedical Research Institute, Department of Immunology and Infection, UHasselt, 3590 Diepenbeek, Belgium; 5 Division of Nephrology, University of Virginia Health System, Charlottesville, VA 22911, USA; ajd9y@uvahealth.org (A.D.R.); mhr9r@uvahealth.org (M.H.R.); 6 1st Department of Nephrology, Transplantation and Internal Medicine, University of Bialystok, 15-540 Bialystok, Poland; jacek.malyszko@umb.edu.pl; 7 Department of Nephrology, Dialysis and Internal Medicine, Medical University of Warsaw, 02-097 Warsaw, Poland

**Keywords:** kidney transplantation, malignancy, renal cell carcinoma, bladder cancer, prostate cancer, penile cancer

## Abstract

Kidney transplantation is the optimal treatment for end-stage kidney failure. However, after successful kidney transplantation, patients require long-term immunosuppression. Due to immunosuppressive therapy, the development of malignancies is more common in solid organ transplant recipients than in the general population. Because renal transplantation has been performed for many decades—much longer than other solid organ transplants—data on malignancies in kidney allograft recipients are the most comprehensive. Malignancies are the third most common cause of death in kidney allograft recipients, after cardiovascular disease and infections. In kidney allograft recipients, malignancies may develop de novo, be transmitted with the transplanted organ, or arise from previously present but undiagnosed cancers in the recipient. Over 90% of malignancies in renal allograft recipients are de novo. In this review, we first present the epidemiology of malignancies after kidney transplantation. Subsequently, the most common urinary tract malignancies, along with their diagnosis, treatment, and screening, are discussed. We also outline the limitations of the published data, propose research priorities, and identify existing gaps and unmet needs.

## Simple Summary

Cancer has become a more common cause of death in patients after kidney transplantation. Patients receiving immunosuppressive therapy, including those after organ transplantation, are more prone to developing malignancies than the general population. In some patients with a history of malignancy, malignancy recurrence may also occur. Among patients who develop neoplasm after kidney transplantation, urinary tract malignancies are the most common. The diagnosis, treatment, and screening of these malignances may differ from those in the general population. Therefore, close collaboration among medical oncologists, urologists, nephrologists, and surgeons is crucial.

## 1. Introduction

Kidney transplantation is commonly performed and currently the preferred method of treating patients with end-stage kidney disease. Following renal transplantation, patients must take immunosuppressive drugs for the rest of their lives or as long as the transplanted organ functions. Immunosuppressive drugs are necessary to prevent rejection, but unfortunately also cause numerous side effects including infections and malignancies.

Since the pioneering work of Dr. Israel Penn (1968–1969), it is known that kidney transplant recipients (KTR) have a significantly higher risk of developing malignancies compared to the general population, a predisposition linked to immunosuppressive therapy [1]. Among solid organ transplants, cancers are the best studied due to the volume and longevity of kidney transplants. Registry data indicate they are the third leading cause of death in KTR, after cardiovascular disease, and their incidence is rising [1,2,3,4]. Mortality attributable to these malignances can reach 13–20% of all deaths in this population [5,6,7,8]. In organ transplant recipients, the following situations can occur: de novo cancers, pre-existing cancers in transplant recipients and tumors transmitted from donor to recipient.

## 2. Methods

Two researchers independently conducted a systematic search of medical databases (Medline, Web of Science, PubMed and Google Scholar) using controlled vocabulary and keywords related to cancer/malignancy/tumor, kidney transplantation (renal transplantation, kidney allograft recipients, kidney transplant recipients), genitourinary tract (renal, kidney, renal cell carcinoma, bladder, urothelial, testicular, testis, penis, penile) and treatment/therapy (surgery, radiotherapy, chemotherapy, immunotherapy, targeted therapy, immune checkpoint inhibitors), screening, and immunosuppression. Following duplicate removal, four independent reviewers screened titles and abstracts, followed by full-text review of potentially eligible studies. Studies were excluded that addressed preclinical data, only those with abstracts, those presented as posters and non-English studies. Disagreements were resolved through discussion or consultation with a third reviewer.

## 3. Incidence of Cancer After Transplantation

An article published in 2020 based on data from the Israel Penn International Transplant Tumor Registry (IPITTR)—the largest registry of patients with cancer after transplantation—reported nearly 10,000 cases of tumor in transplant recipients [2]. The most common malignances observed in patients after organ transplantation were skin cancer followed by post-transplant lymphoproliferative disease (PTLD). The vast majority (over 90%) are de novo cancers in recipients. Malignant neoplasm can occur at any time after transplantation. These findings emphasize the need for ongoing malignancy surveillance in transplant recipients due to their heightened susceptibility, influenced by immunosuppression and other factors.

The latest data from the 47th ANZDATA (Australia and New Zealand Dialysis and Transplant Registry) Annual Report (2024) provides updated figures for malignant disease incidence among KTR [4]. These updated data reflect a continued increase in tumor incidence among long-term transplant recipients, emphasizing the importance of ongoing surveillance and preventive care. Data from registries indicate that urinary tract cancers are relatively rare and are not encountered as frequently after organ transplantation as skin cancers or lymphomas; however, they rank third in terms of incidence [2,4]. Posttransplant malignancy is associated with increased morbidity and mortality [5,6,7,8].

In Taiwan’s National Health Institute Research Database (1997 to 2011) in 5038 kidney transplant recipients (50% living related donors) the most common de novo malignances were transitional cell carcinoma, renal cell carcinoma (RCC) and non-Hodgkin lymphoma [9]. Krynitz et al. [10] reported that in a Swedish population-based study (7952 KTR between 1970 and 2008) the cumulative incidence of cancer was 12% twenty years post-transplantation. In addition, the overall age–sex adjusted standardized incidence ratio (SIR) was the highest for Kaposi sarcoma (KS) (40), followed by vulvar and vaginal (14), lip and oral cavity (10), anal (6.3), and kidney (6.2) cancers. Collet et al. [11] in the United Kingdom (UK) registry examined SIR in 25,104 renal transplant recipients and found that the highest SIR were for lip cancer (65.6), followed by Kaposi sarcoma (17.1), lymphoma (non-Hodgkin lymphoma 12.5, Hodgkin lymphoma 7.4), anal cancer (10.0), and kidney tumor (7.9). In the United States of America (USA) in 35,765 KTR showed a 3-year cumulative incidence of 7.5% for skin and non-skin neoplasms [12]. In the Canadian study the SIR was greatest for non-Hodgkin lymphoma (8.8), oral (7.7), and kidney tumor (7.3) [13]. On the other hand, in the Canadian study by Kitchlu et al. [14] on 325,895 malignancy diagnoses during 29,993,847 person-years of follow-up, bladder tumor, kidney cancers and multiple myeloma were particularly increased in patients with chronic kidney disease (CKD). In addition, mortality rates were negatively associated with impaired kidney function. Higher proportions of stage 4 cancers at diagnosis were more common in patients with CKD. Moreover, KTR had increased risks of cancer-specific death (adjusted hazard ratio (aHR) of 1.48 [95% CI, 1.18–1.87]). In all these studies, the SIR for prostate malignancy was not significantly increased. According to Blosser et al. [15] SIR has remained stable over the last 30 years, except for prostate cancer, for which a significant reduction in SIR was noted in recent years. When comparing the incidence of neoplasms in KTR to patients on waiting lists for kidney transplantation, it was found that transplant recipients have a 39% higher risk of developing kidney tumors compared to those awaiting transplantation [8]. The keynote studies on cancers in renal transplant recipients are presented in Table 1.

## 4. Urinary Tract Malignances—Epidemiology

### 4.1. Kidney Cancer

Among KTR, renal cell carcinoma is the most frequent urinary tract malignancy, affecting 0.5–3.9% of vessels [35,36,37,38]. There are three main types: clear-cell carcinoma-RCC (75%), papillary carcinoma (10%), chromophobe carcinoma (5%), and other types (10%) [39]. Kidney cancer is the most common urinary system malignancy in renal transplant patients, occurring in 0.5–3.9% of kidney recipients [35,36,37,38]. According to the IPITTR registry, it accounts for 4.1% of all post-transplant tumors [2]. The risk of its occurrence in transplant patients is many times (×10–100) higher than in the general population. According to the United States Renal Database (USRDS) from 2024, there is a continued elevated risk of urinary tract malignances among recipients, though specific incidence rates have evolved [3]. Most detected kidney cancers are found in the patient’s own kidneys, and only 9% in the transplanted kidney. It should be remembered that they can occur in the transplanted organ even many years after transplantation. Very rarely, they can be transferred with the transplanted kidney (graft) [40,41]. Such disease is often diagnosed incidentally (e.g., during nephrectomy due to hypertension). Unfortunately, the course of the RCC is more aggressive in KTR than in dialysis patients and the disease has a high mortality rate of about 40%.

Predisposing factors for genitourinary tumors in transplant patients [40,41,42,43,44,45] are the following: abuse of painkillers [42], acquired cysts in the native kidneys, in patients with long dialysis vintage [43,45], and history of prior RCC [40,41]. In addition, older age and male gender are risk factors for native kidney cancer [45].

### 4.2. Prostate Cancer

Prostate cancer is a common malignancy in the general population. In the USA, it is the second leading cause of cancer-related deaths in men with no significant changes in the proportional increase [3]. In KTR, this malignancy is often underdiagnosed and the incidence in organ transplant recipients ranges from 0.3% to 1.9% according to various registries [3,4]. Recently, it has been diagnosed more frequently, likely due to the increasing age of transplant patients [3,4].

According to American data, prostate tumor occurs twice as often in allograft recipients compared to the general population [3], whereas a Swedish nationwide, register-based study showed that kidney transplant patients were not more likely than age-matched control men to be diagnosed with any or high-risk or metastatic prostate cancer [46].

Previous studies did not show a higher incidence in kidney allograft recipients, probably because this population was generally younger [47,48]. Now, as older patients are more frequently transplanted, an increase in prostate malignancy incidence has been observed in this population.

### 4.3. Bladder Cancer

In the general population, bladder malignancy predominantly affects older individuals (60–70 years) [39,49]. It ranks as the fourth most common neoplasm in men (5.5% of all cases) after lung, stomach, and prostate cancer, and the eighth in women (2.3%) [49]. Over 90% of cases are transitional cell carcinoma, originating from the cells lining the urinary tract mucosa [39,49]. Bladder cancer incidence continues to be elevated, approximately three times higher than in the general population in the USA kidney transplant recipients [3]. Moreover, the incidence of bladder tumor is significantly higher in some Asian countries compared to American or European populations [50].

Factors that contribute to bladder cancer have long been known. These include occupational factors (exposure to chemicals such as aromatic amines, dyes, rubber, aniline), lifestyle factors (tobacco smoking), genetic abnormalities and mutations [51,52].

A proven increase in this oncological disease frequency has been observed in patients who received a cumulative cyclophosphamide dose exceeding 20 g [53]. In addition, it has been reported that BK polyoma virus may be a risk factor for development of urothelial bladder cancer in kidney transplant recipients [54,55,56].

### 4.4. Penile and Testicular Cancer

Basile et al. [57] pointed out in their work penile cancer prevalence from 0.04% to 0.3% in their systematic review (in 7 studies out of 21, prevalence of this cancer was reported), with the highest number of 10 cases reported by Besarani et al. [58]. Testicular cancer prevalence ranged from 0.03% to 0.55% in 18 out of 21 examined studies, with the highest number of cases provided by Besarani et al. [58] and Jiang et al. [59] for the systematic review. In only one study, by Piselli et al. [60], SIR was reported to be 4.1 (95% CI 1.3–9.6, *p* < 0.05). The keynote studies on genitourinary malignances are presented in Table 2.

## 5. Symptoms and Diagnosis

### 5.1. Kidney Cancer

Clinical presentation of kidney cancer in transplant patients includes fever, weight loss, feeling of early satiety, anorexia, and hematuria, among other symptoms. It should be noted that many patients are free of symptoms until the disease is very advanced. In some patients, though rarely, the disease may progress rapidly. Kidney tumor metastasizes to lymph nodes, liver, lungs, and invades the renal vein and inferior vena cava. Imaging studies are fundamental in making a correct, early diagnosis. The simplest and easiest imaging study to perform is ultrasound examination. However, renal ultrasound has limited sensitivity when the tumor is small [66]. Computed tomography (CT) and magnetic resonance imaging (MRI) are more sensitive in detecting small tumors. However, these are more expensive tests, and it should be remembered that in patients with impaired glomerular function, complications may occur after these tests such as contrast-induced nephropathy due to contrast agents used in CT. In the diagnostic process, it should be remembered that cytological analysis of urine is of little use, as tumors most often occur in the native kidneys, not in the graft [67,68] and in most KTR the native organs do not produce urine, and only the transplanted kidney produces urine.

### 5.2. Prostate Cancer

The main symptoms include frequent urination, dysuria, urinary retention, hematuria, and bone pain (due to metastases, often to bones). Digital rectal examination (DRE) is a basic, simple examination [69]; however, according to recent data, DRE is not the optimal routine screening test for prostate cancer on its own [70]. The most important laboratory test is Prostate-Specific Antigen (PSA). Using both tests seems to be valuable in making a correct diagnosis [71]. Other tests used include transrectal ultrasound, CT, and MRI. Prostate biopsy in transplant patients is as safe as in the general population [72]. Patients should receive prophylactic antibiotics (fluoroquinolone) before biopsy. Recently, Whang et al. [73] proposed genomic testing in addition such as Oncotype DX, Decipher or Polaris.

### 5.3. Bladder Cancer

The main symptom of bladder cancer is hematuria, which is usually painless and accompanied by blood clots. Other common symptoms include frequent urination and dysuria. These symptoms may suggest urinary tract infection, but if urine culture is negative and symptoms persist, further tests for bladder malignancy should be conducted. Diagnosis involves ultrasound, cystoscopy, urography, and CT [74]. Cytological diagnostics can be helpful in diagnosing lower urinary tract tumors.

### 5.4. Penile and Testicular Cancer

Penile cancer symptoms include an abnormal growth on the penis, rash, or sore that does not heal, bleeding, changes in skin color or thickness, and discharge under the foreskin. Testicular cancer symptoms often involve a lump or swelling in a testicle, a feeling of heaviness in the scrotum, or a dull ache in the lower abdomen or groin. Some testicular malignances may not cause any symptoms, making regular self-exams important. Diagnosis of penile and testicular tumors begins with a physical exam, followed by imaging tests like ultrasound to confirm masses. For penile cancer, a tissue biopsy is necessary for a definitive diagnosis. For testicular cancer, if an ultrasound is positive, an inguinal orchidectomy is often performed to obtain tissue for a definitive pathological diagnosis. Further diagnostic steps often include blood tests, especially for testicular malignancy markers (beta-human chorionic gonadotropin, alpha-fetoprotein and lactate dehydrogenase), and imaging, such as CT scans, to check for metastases.

## 6. Treatment

### 6.1. Surgery

In general surgery is the first line option for genitourinary tract malignances. Surgical treatment is central in the management of kidney cancer, with good results when implemented early in the disease course. In advanced forms, results are unfortunately much worse [75]. Therefore, as with other malignances, early diagnosis is crucial for a better prognosis.

When a tumor is in the transplanted kidney, graftectomy and return to dialysis may be necessary. Nephron-sparing surgeries in cases of non-metastatic cancers smaller than 4 cm, located peripherally, have been described in only a few cases. Radiofrequency ablation and cryoablation could also be considered [76]. Thus, localized RCC in KTR is treated similarly to the non-transplanted population [77]. When metastases are present in KTR, immunosuppression should be discontinued, nephrectomy performed, and immunotherapy initiated [78]. The most common treatment for prostate cancer is a radical prostatectomy, the same as in the general population [79]. Removal of lymph nodes on the graft side can also be problematic during prostatectomy. For bladder cancer, treatment is the same as in the general population, primarily involving surgical intervention such as transurethral resection of bladder tumor (TURBT) for low-risk non-muscle invasive tumors. In the recent review by Grabe-Heyne [80], treatment options for non-muscle-invasive bladder cancer (NMIBC), with its burden and limitations, are presented. Intravesical Bacillus Calmette-Guérin (BCG) is an adjunctive therapy to the surgery in intermediate and high-risk patients to reduce recurrence and progression [81,82]. An alternative to BCG is intravesical chemotherapy in patients with intermediate-risk NMIBC, whereas in high-risk patients, when BCG is not available or not tolerated, other options include mitomycin C, epirubicin, docetaxel or gemcitabine [83]. In muscle-invasive tumors, the gold standard is radical cystectomy. In kidney transplant recipients, together with radical cystectomy, limited pelvic node dissection, urinary diversion with eventual bilateral nephrouretectomy is the preferred method of treatment [84]. Primary penile and testis malignances are treated surgically (penectomy or orchidectomy, respectively) as in the general population. In the recent narrative review, Dell’Atti and Slyusar [85] stressed that the treatment of locally advanced disease posed unique challenges due to the presence of the graft, regarding both chemotherapy (mainly cisplatin-based regimen) and radiotherapy. However, in localized disease, non-cytotoxic alternatives should be prioritized when feasible. They also underlined limited evidence available on therapy of penile and testicular cancers in this population. Therefore, deviations from established treatment guidelines are to be justified [57,85].

### 6.2. Radiotherapy

Radiotherapy is not used to treat renal cancer and is limited to exceptional cases; however, it may be a viable option if the neoplasm is still only in the kidney, but a person is not healthy enough for (or does not want to undergo) surgery or has a solitary kidney (dialysis after the surgery is also an option to be discussed) [86,87]. The technique used is called External Beam Radiation Therapy (EBRT). However, when treating the tumor in the kidney or a small area of cancer spread (such as single lung metastasis), radiation is usually given as a stereotactic body radiation therapy (SBRT) known also as a stereotactic ablative body radiotherapy (SABR).

In bladder cancer, radiotherapy can be used as part of the treatment for some early stage of this disease when surgical treatment was not radical in removing the whole bladder (such as TURBT), as part of the treatment for people who cannot have (or do not want) a cystectomy, as part of treatment for advanced bladder tumor or as in other cases to help prevent or treat symptoms caused by advanced kidney/bladder or prostate malignancy as palliative therapy. In case of bladder cancer, radiotherapy is often combined with chemotherapy (with cisplatin, gemcitabine, capecitabine, or 5-fluorouracil (5-FU) plus mitomycin; these two latter therapies are not used in the treatment of urothelial malignancy) as chemoradiation to enhance the radiation therapy efficacy [88,89].

Radiotherapy may be used as the only treatment for prostate cancer, or it may be used with other treatments, such as chemotherapy or surgery [90,91]. It is a common and effective treatment for all stages of the disease and can be delivered either externally (EBRT) or internally (brachytherapy).

In KTR, there are some specific limitations and difficulties in treatment as the graft is located close to the prostate gland, and radiotherapy can cause radiation nephritis. Shielding of the transplanted kidney during radiotherapy is recommended. In the case of penile or testicular tumor, prophylactic radiotherapy to retroperitoneal nodes can present a problem with injury to allograft in the pelvis and the development of retroperitoneal fibrosis.

### 6.3. Hormone Therapy

Hormone therapy, also called androgen deprivation therapy, lowers or blocks testosterone [90,91], and is used for treatment of prostate cancer.

### 6.4. Chemotherapy, Targeted Therapy and Immunotherapy

Kidney cancer typically does not respond well to classical chemotherapy, while targeted therapy and immunotherapy are the most common treatments for most advanced kidney malignances. Treatment of kidney cancer in the allograft does not differ from the general population and includes either a combination of 2 immune checkpoint inhibitors (ICI) of different modes of action (i.e., Programmed Death-1 (PD-1) inhibitor—nivolumab and cytotoxic T lymphocyte-associated antigen-4 (CTLA-4) inhibitor—ipilimumab, or a combination of a checkpoint inhibitor with a tyrosine kinase inhibitor (TKI), for example, pembrolizumab and axitinib or cabozantinib with nivolumab. These combinations administered as a first line treatment in metastatic RCC showed superiority in progression-free survival, objective response rate, and overall survival when compared to monotherapy with TKI, the previous standard of care [92,93,94]. In 2025, Updated European Association of Urology Guidelines on the Use of Adjuvant Immune Checkpoint Inhibitors and Subsequent Therapy for Renal Cell Carcinoma, adjuvant use of pembrolizumab, PD-1 inhibitor receives a strong recommendation [95]. For advanced RCC, there is currently no trial evidence for the optimal immunosuppression regimen or oncological treatment in kidney and/or solid organ transplant recipients.

For prostate cancer, most often, docetaxel is the first chemotherapeutic drug given, together with steroids (followed by cabazitaxel along with a steroid) if docetaxel does not work or stops working. Other options include mitoxantrone, estramustine, and carboplatin [90]. Pembrolizumab in the first line, followed by cabazitaxel in the second line is the new standard [91].

Chemotherapy for bladder cancer can be given in 2 different ways, either intravesically (for early stage usually given within 24 h of the TURBT) or systematically in neoadjuvant settings (before surgery) or after surgery or sometime after radiotherapy in adjuvant settings. Considering choice of chemotherapy, we have to consider first of all graft function to adjust the dose of the drug or look for an alternative [96]. Immunotherapy or antibody–drug conjugates, i.e., enfortumab vedotin or plus pembrolizumab in first line is the new gold standard. Sacituzumab govitecan, only in subsequent lines [88,89] may also be an option depending on the indications and availability.

For penile and testicular malignances, chemotherapy has been reported as an option in selected cases. A few reports described the use of chemotherapy in transplant recipients for the management of metastatic diseases [97]. In KTR, the choice and dosing of chemotherapeutic agents should be carefully determined considering graft function and potential drug–drug interactions. Increased allograft rejection rates have been reported with several chemotherapeutic agents and especially ICI [98,99,100,101,102]. Particular attention should be paid to possible adverse events such as thrombotic microangiopathy after treatment with gemcitabine or anti-angiogenic therapies [103]. It is of paramount importance as calcineurin inhibitors (CNI), an essential part of the immunosuppressive regimen, predispose to endothelial injury and development of this life-threatening complication.

In a few studies on organ transplant recipients, ICI were mainly used in skin cancer, malignant melanoma and non-small cell lung cancer [95,99,100,101,102]. In the French study of Legris et al. [104], 34 patients after kidney transplantation were analyzed, none of whom had genitourinary tract malignancy. Use of ICI was associated with a 38% tumor response rate and a 15% incidence of graft loss. The major limitation of the studies on ICI use in organ transplant recipients is the lack of data on genitourinary tract malignances; only in the study by Murakami et al. [101], 3 kidney and 2 bladder tumors among 69 transplant recipients were analyzed. In addition, data on the outcomes, graft failure and deaths were mainly analyzed. It has been reported that in solid organ transplant recipients with malignancy, treatment with ICI either in monotherapy or in combination has been associated with an increased risk of rejection [105,106,107,108]. It was also reported that use of ICI may cause acute kidney injury [109,110], which can manifest in renal transplant recipients as worsening of graft function. In solid organ transplant recipients, except kidney, acute rejection not responding to therapy leads to a fatal outcome, whereas in renal allograft recipients, kidney replacement therapy in the form of dialysis is a viable backup option in case of graft loss. A possibility of evaluation for a retransplantion is another option upon curative cancer treatment. Therefore, personalized approach, individual risk–benefit assessment with a multidisciplinary team. is of paramount importance. In particular—in the setting where there are no definitive recommendations due to the lack of good large clinical trials, and very limited data available. Moreover, the 34th Acute Disease Quality Initiative meeting held in Charlottesville in 2024 aimed to develop a Consensus Statement to present guidance on the prevention, diagnosis and management of anti-cancer therapy nephrotoxicity. Finally, “The nephrotoxic effects of anti-cancer therapies: consensus report of the 34th Acute Disease Quality Initiative workgroup” was published in *Nature Review Nephrology* in December 2025 [111]. The consensus panel stated that kidney allograft recipients, being at increased risk of renal complications of ICI, required careful assessment of the risks and benefits of treatment with these drugs, along with possible immunosuppression adjustment and close monitoring of graft function. In addition, the panel also acknowledged the shared decision to initiate ICI in kidney allograft recipients considering duration of transplantation, immunization, i.e., presence of donor-specific antibodies, previous rejection, availability of alternative anti-cancer therapy as well as patient preferences (weighing the risk of inferior neoplasm outcomes versus the risk of returning to dialysis). The panel also discussed the role of steroids (increasing dose or restarting) before ICI initiation together with switch from CNI to mammalian target of rapamycin (mTOR) inhibitors [111].

## 7. Immunosuppression in Kidney Transplant Patients with Malignances

The issue of immunosuppressive treatment in patients with genitourinary tract malignances is extremely important. Unfortunately, due to the relatively small number of these malignances in patients after transplantation, there is a lack of large studies and, consequently, recommendations. The general approach, although without evidence, is reduction in immunosuppression, rather than withdrawal of immunosuppressive therapy. The assumption is made on the basis that malignancy development after kidney transplantation is due to the cumulative burden of immunosuppression [112]. This is the primary approach for graft recipients, as even the graft loss is not a fatal event as in the case of other solid organ transplantation. The optimal approach to reduce immunosuppression is still debatable/uncertain. Discontinuation of antimetabolite rather than withdrawal of CNI is the preferred strategy. Switching from CNI to mTOR is rather not recommended due to the adverse impact on overall survival [113,114]. Switching to an mTOR inhibitor and prednisone may also be an option; however, no data support this approach. In very well-matched human leukocyte antigen (HLA) transplant recipients (0-HLA or 0-B, 0-DR mismatches), with low risk of rejecting the combination of an antimetabolite and prednisone could be a viable option. In these patients, CNI withdrawal rather than the antimetabolite appears reasonable as nephrotoxicity and malignancy potential associated with a CNI is to be minimized. When ICI is to be introduced, the maintenance immunosuppression regimen should be adjusted, bearing in mind the worsening of graft function and/or rejection, although the optimal approach to this is not known. Combination of low-dose tacrolimus (target trough level 3 to 5 ng/mL) and steroids may be considered; however, Schenk et al. [115] in one small trial in kidney transplant recipients with advanced skin cancers found that this combination did not provide adequate protection against allograft rejection and compromised immune-mediated tumor regression. Data on efficacy and safety of immunosuppression reduction in renal allograft recipients are scarce and based mainly on observational studies, case reports and case series. The same applies for immunosuppression and genitourinary malignances, where data are even more limited. Pommerolle et al. [43] suggested that modification of immunosuppressive treatment should be discussed in patients with RCC.

## 8. Screening

### 8.1. Kidney Cancer [12,39]

**Figure 1:**
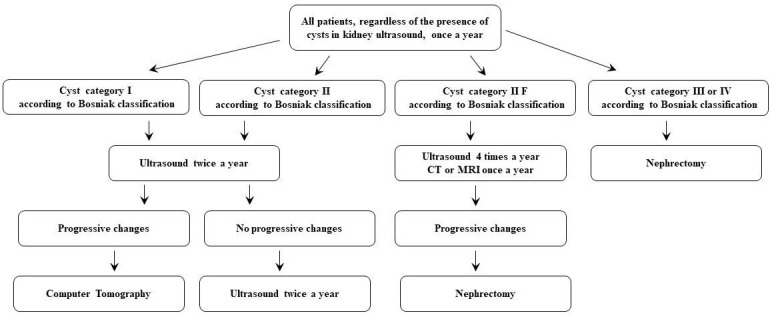
Screening algorithm and management in the patients with kidney cysts.

Compared to the general population, KTR at the time of renal cancer diagnosis are younger (47 vs. 62 years), while the stage at diagnosis and 1-year survival rates did not differ between the populations. According to the authors of this study, immunosuppression has a relatively small impact on short-term survival of transplant patients with kidney neoplasm compared to the general population. A Japanese study [116] also found good survival rates for renal cancer patients, with 67% of patients surviving for five years. However, a limitation of this study, as well as many others in the post-transplant population, is the small number of cases. Recently, updated, less restrictive guidelines regarding the suitability of oncological patients, including urinary tract malignancies for the transplant waiting list, were reported by the Kidney Disease: Improving Global Outcomes (KDIGO) (2020) (Table 1 and Table 2) [117,118], and the American Society of Transplantation (AST) (2021) (Table 2 and Table 3) [119,120]. Cancer screening and treatment in KTR were nicely reviewed in 2024 by Bigotte-Vieira et al. [121]. AST and KDIGO guidelines do not recommend screening for RCC [122,123,124]. The European Association of Urologists recommends annual evaluation, and the European Best Practice Guidelines group recommends regular RCC screening in the native organs using ultrasonography, with no specification about frequency [125,126,127]. In 2007, a German group published a study involving 561 KTR in whom ultrasound examinations revealed the presence of kidney cancer in 4.8% of patients, and in those with acquired cysts, the rate was as high as 19.4% [67] (Figure 1). It seems that these recommendations are reasonable and should be applied to kidney transplant patients. In addition, kidney transplant recipients with a family history of RCC, familial syndrome, and acquired cystic disease (ACKD) should be screened for RCC on an individualized basis [128,129]. As stressed by Dahle et al. [77], screening for RCC in all KTRs is not cost-effective but may be of value in high-risk subsets, such as patients with a previous RCC and patients with known ACKD. Ultrasonography, despite being operator-dependent, is the preferred method of screening. However, CT remains a gold standard for diagnosis in acquired kidney disease, despite higher costs, risk of radiation, and post-contrast acute kidney injury [129].

Recently, since risk factors and the value of screening remain unclear for RCC, Pommerolle et al. [43] conducted a multicenter case–control study comparing annual screening for RCC with other strategies (either ultrasound at 1 year of KTx then every 3 years or no systemic screening). They found that annual screening could improve outcomes (fewer relapses and lower rate of mortality due to malignancy). However, they stressed that their findings should be evaluated in larger studies and special high-risk groups (males and patients with ACKD) should have priority for screening.

### 8.2. Prostate Cancer

There are no specific guidelines for prostate cancer screening in KTR. American guidelines for transplant recipients recommend that patients over 50 years old should have a DRE and PSA test once a year if their expected survival is 10 years. For men between 40 and 50 years old, these tests are recommended for selected high-risk patients (African Americans, family history of the disease) [119,130]. Vitiello et al. [131] retrospectively analyzed the effect of PSA-based screening on transplantability. They found that patients screened based on PSA (positive PSA screening with level more than >4 ng/mL) not only had a lower chance of receiving a transplant, had a longer time to be actively waitlisted, but also had no improved survival after transplantation. On the basis of their findings, Vitiello et al. [131] were not in favor of PSA screening for prostate malignancy in KTR.

### 8.3. Bladder

There are no screening tests (urine tests) for asymptomatic kidney transplant patients for urinary tract cancers. However, annual screening with cystoscopy and cytological examination is recommended for patients with non-glomerular hematuria who were previously treated with cyclophosphamide [132,133,134]. Additionally, screening may be necessary for patients who previously abused painkillers, Chinese herbs, or were treated with cyclophosphamide. American centers recommend the following for patients with hematuria or at risk: urine assessment every 3–6 months, urine cytology (repeated 3 times), ultrasound of native kidneys, graft, and bladder, PCR for BK polyoma virus, and PSA test.

It is crucial to include bladder examination during routine ultrasound scans for transplant patients, especially those with a history of cyclophosphamide treatment, painkiller use, or Chinese herb consumption.

No data are available on penile and testicular cancer screening as they are very rare in KTR.

## 9. Cancers Transmitted Through Transplantation

Cancer cells can be transferred along with a transplant when an undiagnosed tumor is present in the organ donor. While these cases are sporadic, it is important to keep this possibility in mind. Therefore, donors with a history of malignancy should be excluded (except for non-metastatic brain tumors). Currently, cases of tumor transmission through transplantation are very rare and do not pose a significant problem [36,37]. According to American data from the United Network for Organ Sharing (UNOS) from 1994 to 2001 (34,933 donors, 108,062 recipients), only 21 cases of cancer transmission from donor organs were reported [37]. There were also 8 deaths among such patients out of 108,062 recipients (0.007%). The authors of this report conclude that the risk of neoplasm transmission from a donor organ and subsequent death is exceptionally low, especially when compared to the mortality rate of patients on the transplant waiting list. The most reported transmitted malignances include primary brain tumors, kidney cancer, lung cancer, malignant melanoma, choriocarcinoma, liver, bile duct, and breast cancer [37]. Based on the data obtained so far, among urinary system malignances, only renal tumor is considered a potential risk for transmission through transplantation.

## 10. Pre-Existing Cancers

The presence of cancer is, of course, a contraindication for kidney transplantation. However, many patients on dialysis and waiting for a transplant have previously had malignances [23,135,136,137,138]. In our recent study, among 5879 prevalent hemodialysis subjects (60% men), 757 had a history of malignancy. In this population, 449 patients were actively waitlisted, including 27 patients with a prior cancer, representing 3.8% of the patients studied [138].

Studies have shown that the recurrence rate in patients with a history of cancer ranged from 22 to 27% [139]. Patients with previous oncological history are also at increased risk of de novo cancers after kidney transplantation [140].

Significant differences were observed depending on the type of previous neoplasm as shown in Table 3. These data indicate that urinary tract cancers can recur and do so quite frequently.

The high frequency of cancer recurrence has forced us to assess the risk of recurrence of past malignancy in transplant recipients. The difficult question “When can a transplant be safely performed?” depends primarily on the type of cancer. Different waiting periods are recommended for various types of neoplasm (and stage and aggressiveness), usually ranging from 2 to 5 years [80,82,84,101].

Therefore, a decision concerning waiting time should be discussed with an oncologist on an individual basis. For such patients, after kidney transplantation, it is certainly necessary to avoid aggressive immunosuppression and consider use of mTOR inhibitors. It is probably better to discontinue calcineurin inhibitors as quickly as possible or use them with low target trough levels. Unfortunately, there are no definitive recommendations due to the lack of good large clinical trials.

**Table 3:** Recurrence rate of malignancy after kidney transplantation [139,140].

Recurrence Rate After Transplantation (%)	Cancer Type
0–10%	• localized kidney cancer • testicular cancer • cervical cancer • thyroid cancer
11–25%	• Wilms tumor • colon cancer • prostate cancer • breast cancer • uterine cancer
>25%	• bladder cancer • advanced kidney cancer • sarcoma • multiple myeloma • skin cancers

## 11. Counseling About Individual Cancer Risk and the Importance of Screening Regarding Urinary Tract Cancers

First, counseling about individual cancer risk and the importance of screening is critical and should be done at the time of evaluation for renal transplantation (ideally at low clearance clinic or outpatient nephrology care).

In the conversation, increased risks, the importance of pre-transplant screening for latent malignances, and tailored post-transplant screening schedules for all neoplasms, including urinary tract malignances, should be covered. (for kidney cancer, i.e., repeated sonography of native kidney/s, if something abnormal on sonography, proceed with further imaging such as CT with contrast).

Explanations about increased tumor risk due to long-term immunosuppression, necessary to prevent graft rejection also weakening the immune system’s ability to fight cancer cells, pre-transplant history of malignances, and age, should be given to candidates/renal transplant recipients.

A personalized screening plan after the transplant is to be presented for recipients with prior neoplasm (in particular urinalysis, ultrasonography of native kidneys/renal allograft, bladder, PSA, on regular basis at least annually).

Patient-specific considerations should be brought up such as harms vs. benefits (i.e., cancer screening has potential harms, such as false positives, and that the benefits must be weighed against the risks for each individual), potential chances for recurrence for the patient with prior malignancy (i.e., importance of monitoring for recurrence and the need for potential adjustment of immunosuppression, for the price of potential enhanced risk of graft rejection) and symptom awareness (patients to be encouraged and vigilant about any new or unusual symptoms to report them immediately, i.e., hematuria, flank pain).

## 12. Limitations of Published Data, Research Priorities and Unmet Needs

Epidemiology data on de novo cancers in kidney transplant recipients are generally retrospective, single-center, with no abundant number of patients relative to epidemiological studies on malignancy prevalence in the general population. In addition, data are mainly derived from North America [5,13,14,15], Australia and New Zealand [4,141], and Northern and Western Europe [7,10,11,142]. Studies from other continents such as Africa, South America, different parts of Asia or Central-Eastern European countries are limited. Some of them are historical today [143,144], and substantial number of papers were published more than 10 years ago, and their relevance today could be questionable.

Retrospective design has certain bias, in particular missing or incomplete data, heterogeneity, different immunosuppressive regimen, immunosuppressive treatment/cumulative immunosuppression before transplantation, different characteristics of donor and recipient (extended criteria donors, non-heart beating donors), prevalence of rejection episodes and treatment, different rates of BK virus and cytomegalovirus infections, year of transplantation, dialysis vintage, HLA-matching, number of transplantation, history of malignancy prior to transplantation and its stage and therapy. Many of these data are unavailable, in particular in patients undergoing renal transplantation many years ago. There is a lack of prospective studies. There are also discrepances in available guidelines and updates would be of value [111,120,121,122,145,146].

Taking all these into consideration, we propose research priorities (Table 4) and stressed unmet needs and knowledge gaps from both perspectives: oncology and nephrology (Table 5).

**Table 4:** Research priorities in urinary tract malignancy after kidney transplantation.

	**Research Priorities**
Epidemiology	• generate observational data of good quality • unify coding (ICD-O) • merge and harmonize transplant data with oncology sources • capture granular data on histology, stage, and outcomes • establish global registry of good quality (kidney replacement therapy including transplantation)
Recipients with history of malignancy	• establish observational data of good quality • merge data with oncology sources • immune profiling and genomic profiling
Cancer screening in KTx	• enhance malignancy screening across major nephrology and transplant centers and report data • perform an intervention trial for RCC screening in the at-risk population globally
Malignancy management after KTx	• create/merge cancer registry with transplant registry • develop malignancy treatment protocol • industry to be involved • enroll patients to RCT with the new cancer therapies in KTR, including those with low GFR • conduct RCT to assess mTORs versus other immunosuppressive regimens in cancer patients after Ktx • create exchange platform to share data

CKD—chronic kidney disease; GFR—glomerular filtration rate; ICD-O—International Classification of Diseases for Oncology; KTR—kidney transplant recipients, KTx—kidney transplantation; mTOR—mammalian target of rapamycin; RCT—randomized controlled trials.

**Table 5:** Knowledge gap and unmet needs in transplant oncology.

Oncology Perspective	Nephrology Perspective
huge burden of patients	transplant recipients are under care of transplant physicians/general practitioners/nephrologists—fragmented care
limited human resources	not every nephrologist takes care of transplant patients
patients with impaired kidney function, in particular kidney transplant recipients are very challenging	very limited data on malignancy in kidney transplant recipients
lack of/limited access to nephrology consults	limited/lack of access to oncology consults
lack/limited dialysis support in some cancer centers	dialysis support is challenging in oncology patients
problems with nephrology referral	problems with oncology referral
lack of expertise on nephrology treatment	lack of expertise in oncology treatment
prejudice on contract use for imaging studies, CT with contrast difficult to perform on ambulatory basis	
patients after kidney transplantation—issues with drug dosing, alternate therapies, contraindications, severe adverse events, limited treatment option due to impaired kidney function, drug interactions	
lack of specialists with expertise in onconephrology/nephrooncology	
lack of data on patients with impaired renal function/after kidney transplantation,	
exclusion of patients with impaired kidney function/after kidney transplantation from oncology trials	
data coming from case series, case studies, no RTCs	
very demanding, selected population with multimorbidities	
different data collected depending on the specialty, data from cancer registries on the first course of cancer treatment, type of cancer, data from transplant registries mainly on cancer prevalence	
data mainly from retrospective cohort studies	
lack of/very limited detailed longitudinal cancer treatment information in kidney transplant recipients	
the evidence for robust cancer screening pre- and posttransplantation is limited	

CT—computed tomography, RTC—randomized controlled trial.

## 13. Conclusions

Cancers are the third most common cause of death in KTR. In addition, urinary tract malignances became the third most common neoplasm after kidney transplantation. In addition to modifying immunosuppression regimen, KTR are eligible for most cancer-targeted therapies, including surgery, cytotoxic chemotherapy and radiation. Moreover, current recommendations on screening and therapy are still largely based on the data from the general population, but their validity is uncertain in KTR. All data collected from registries, cohort, retrospective studies, etc., are shared and analyzed to develop standardized screening guidelines for malignancy posttransplant. This is not only clinically relevant but of paramount importance for highly prevalent cancers after transplantation including urinary tract malignancies. In the most recent systematic review, the incidence, clinical presentation, treatment and survival outcomes of upper urinary tract, in KTR based on 16 retrospective studies including 526 patients were presented [147]. In our narrative review we also included lower urinary tract malignances, epidemiology, screening, and immunosuppression.

In conclusion, the approach to posttransplant malignancies begins with general preventive measures. Careful screening of the patient and donor prior to transplantation to help detect an underlying, preexisting malignancy should also be performed to yield the best possible outcomes after transplantation.

## Figures and Tables

**Table 1:** Keynote studies on malignancy in transplant recipients, methodology and main findings.

	Country	Population	Study Design	Malignancy	Main Findings
1. Taborelli et al. [16]	Italy	13,245 KTR	retrospective matched cohort	all	Cancer was associated with greater risk of mortality with functioning graft (HR = 3.31), for kidney cancer (HR = 2.38)
2. Taborelli et al. [17]	Italy	7373 KTR	retrospective cohort	all	Malignancy is a leading cause of death (32.4%), kidney cancer (SMR = 5.44, 95% CI: 2.97–8.88)
3. Laowalert et al. [18]	Thailand	1156 KTR	retrospective cohort	all	Urothelial cancer—6.9 per 1000 person-years (age-and sex-adjusted incidence rate after Ktx), SIR—42.5, kidney cancer—SIR of 24.4 association of cancer with death, but not graft failure
4. Cheung et al. [19]	Hong Kong	4895 KTR	retrospective population-based cohort	all	Kidney cancer SIR of 12.5 (8.51–18.36), bladder cancer—8.22 (4.67–14.47), prostate cancer—0.88 (0.39–1.95). Mortality rates for kidney and bladder cancer higher in transplant recipients over general population
5. Li et al. [20]	Taiwan	4716 KTR	nationwide population-based cohort study	all	Kidney cancer SIR of 44.29, bladder cancer SIR of 42.89, age less than 20 years at transplantation associated with the highest risk of post-transplantation cancer
6. Tsaur et al. [21]	Germany	1990 KTR	retrospective	all	Urological malignances—3.3% cumulative incidence and most common mortality cause—(44.4%)
7. Jung et al. [22]	Korea	12,634 KTR	retrospective nationwide population-based cohort study	all	aRR for kidney cancer—14.9; 95% CI, 7.9–28.3, bladder/urinary tract—4.1; 95% CI, 2.3–7.3. Higher risk for kidney cancer males and females, whereas only in females, bladder/urinary tract cancers had higher relative risk
8. Oliveras et al. [23]	Catalan/Spain	8037 KTR	population-based study	all	Kidney, bladder and urinary tract cancers had 3–5 higher risk than expected, whereas only prostate cancer was lower risk than expected
9. Ietto et al. [24]	Varese, Italy	462 KTR	retrospective cohort	all	Urinary tract cancers together with non-melanoma skin cancers, Kaposi sarcoma, hematological tumors were the most common. SIR of 2.8; 95% CI 1.8–4.3 for all cancers in kidney transplant recipients
10. Nimmo et al. [25]	Scotland	4033 KTR	nationwide cohort study	all	Non-melanomatous skin cancer (57.9%), lymphoma (7.9%), kidney (7.0%), lung (6.8%), breast (4.0%), and prostate (3.7%) cancer are the most common. SIR of 9.4 [IQR 4.2–20.9] in females and 12.2 [IQR 8.0–18.5] in males aged 45–59 years. SIR of 3.1 [IQR 1.1–8.2] in females, SIR 5.0 [IQR 3.2–8.0] in males over 60 years of age. Bladder cancer had higher incidence of stage 4 metastatic disease
11. Srisuwarn et al. [26]	Thailand	2024 KTR	retrospective cohort single center	all	Urothelial cancer most common, (20% of all cancers) with an SIR of 114.7 (95% CI 66.8, 183.6) in females. The SIR for prostate cancer 8.11 (95% CI 3.71, 15.4)
12. Komorowska-Jagielska et al. [27]	Poland	246 KTR	retrospective cohort 2 centers	all except non-melanoma skin cancer	The most prevalent cancers in males were kidney (16.4%), lung (15.7%), and prostate (14%), in females, breast cancer led (17.8%), followed by colon (14.5%), lung, and PTLD (8.9% each). Notably, native kidney cancer was highly frequent, with 5.8% of transplant recipients having a history of prior malignancy
13. Lizakowski et al. [28]	Poland	3069 KTR	retrospective cohort 2 centers	all	Gastrointestinal tract (25%), urinary tract tumors (23.2%), lung cancer (n = 18; 16%), and lymphoma (13.4%) were the most common
14. Kalil et al. [29]	USA	109,224 primary KTR and 6621 re KTR	retrospective cohort	all	In retransplants only renal/pelvis cancers had a higher incidence, in particular RCC (IRR 1.72, 95% CI 1.24–2.31, *p* = 0.0007), whereas prostate cancer had lower incidence in retransplants (IRR 0.61, 95% CI 0.37–0.94)
15. Gioco et al. [30]	Catania, Italy	535 KTR	retrospective cohort	all	After Ktx native kidney cancer was diagnosed in 13%, the most common was Kaposi sarcoma in 23%
16. Krishnan et al. [31]	Australia, New Zealand	21,844 KTR	retrospective cohort	all	De novo cancers in 13%, with PTLD (25%), urinary tract cancers (18%) and malignant melanoma (12%) diagnosed early (<12 months after Ktx). PTLD (14%), urinary tract cancers (13.6%) and melanomas (10.6%) diagnosed late (more than 1 year after Ktx)
17. D’Arcy et al. [32]	USA	7,147,476 general population 11,416 SOT with cancer	retrospective cohort	all	Cancer-specific mortality was higher in solid organ transplant recipients, in particular for. melanoma, breast and bladder cancers (1.85, 1.58, 2.17)
18. Farrugia et al. [33]	UK	19,103 KTR	retrospective cohort	all	Cancer was cause of death in 7.4%. Lymphoma (18.4%), lung (17.6%) and renal (9.8%) were the three most common. Cancer-related deaths were more common in the 2006–2012 versus 2001–2006 era
19. Mazzucotelli et al. [34]	North-western Italy	735 KTR (75 with cancer) 912 dialysis (42 with cancer)	retrospective	all, 57 solid cancers, 13 PTLD, 12 KS, in dialysis, 42 solid cancers	After Ktx—2.1-fold rise in cancer risk induction therapy associated with a significant increased risk of KS; virus-related cancers only diagnosed after post-transplant

SOT—solid organ transplant, KTR—kidney transplant recipients, Ktx—kidney transplantation, PTLD—post-transplant lymphoproliferative disease, KS—Kaposi sarcoma, SMR—standardized mortality ratio, SIR—standardized incidence ratio, HR—hazard ratio, CI—confidence interval, IQR—interquartile range, aRR—adjusted risk ratio, IRR—incidence rate ratio.

**Table 2:** Keynote studies on genitourinary tract malignances in kidney transplant recipients, methodology and main findings.

	Country	Population	Study Design	Malignancy	Main Findings
1. Jiang et al. [59]	US, University of Minnesota, USA	6172 KTR	cohort	genitourinary (kidney graft and native, prostate, bladder, penile, testicular)	The most common malignances were RCC (native and transplanted kidney) prostate and bladder. Higher incidence of RCC, lower incidence of prostate, and comparable incidence of bladder cancer relative to the national age-matched population
2. Antunes et al. [61]	Portugal	2897 KTR	retrospective single-center	urologic malignancies	Among all, 2.2% had urologic malignancies. The overall 5-year survival rate was 82.8%. Tumor-related death was in 13.8%, while graft loss in 1/3 of the patients
3. Elkentaoui et al. [62]	France	1350 KTR	retrospective study	urologic malignancies	Among all, 3.1% had urologic malignancies
4. Kim et al. [63]	Korea	31,542 KTR	national cohort	urologic malignancies	Urinary malignances have increased risk in KTRs, prostate cancer risk comparable to the general population
5. Minkovich et al. [64]	Canada	2443 KTR	observational cohort study	renal cell carcinoma-RCC	RCC had higher risk in KTR relative to general population, found in 2.1% of all KTRs, mainly within 4 years after Ktx. Predominant stage—T1a in 86.3%. clear-cell type diagnosed in 45.1%, RCC found in the native kidney in 80.4% was associated with higher incidence of other malignancies in KTR
6. D’Arcy et al. [65]	USA	262,455 SOT	retrospective cohort	33 rare cancers, with 694 subtypes, including kidney, bladder, penile	The most common types of RCC were clear-cell, papillary and renal cell adenocarcinomas. Strong associations (SIR = 10–19 range) between bladder micropapillary transitional cell carcinoma and signet ring cell adenocarcinoma; as well as kidney papillary adenocarcinoma and collecting duct carcinoma. More modest associations (SIR = 2.2–10) were observed for bladder (adenocarcinoma NOS, penis, SCC, RCC chromophobe type). Increased incidence of papillary adenocarcinoma after 5 years post-transplant

SOT—solid organ transplant, KTR—kidney transplant recipients, Ktx—kidney transplantation, PTLD—post-transplant lymphoproliferative disease, KS—Kaposi sarcoma, SMR—standardized mortality ratio, SIR—standardized incidence ratio, HR—hazard ratio, CI—confidence interval, NOS—not otherwise specified, SCC—squamous cell carcinoma.

## Data Availability

No new data were generated during the study.

## References

[1] Peen I., Hammond W., Brettschneider L., Starzl T. (1969). Malignant lymphomas in transplantation patients. Transplant. Proc..

[2] Penn I. (2000). De novo cancers in 9688 renal allograft pts. Adv. Ren. Replace Ther..

[3] 2024 Annual Data Report. https://usrds-adr.niddk.nih.gov/2024.

[4] ANZDATA Australia and New Zealand Dialysis and Transplant Registry. https://www.anzadata.org.au.

[5] Wang J.H., Pfeiffer R.M., Musgrove D., Castenson D., Fredrickson M., Miller J., Gonsalves L., Hsieh M.-C.P., Lynch C.F., Zeng Y. (2023). Cancer Mortality Among Solid Organ Transplant Recipients in the United States During 1987–2018. Transplantation.

[6] Acuna S.A., Fernandes K.A., Daly C., Hicks L.K., Sutradhar R., Kim S.J., Baxter N.N. (2016). Cancer Mortality Among Recipients of Solid-Organ Transplantation in Ontario, Canada. JAMA Oncol..

[7] Friman T.K., Jäämaa-Holmberg S., Åberg F., Helanterä I., Halme M., Pentikäinen M.O., Nordin A., Lemström K.B., Jahnukainen T., Räty R. (2022). Cancer risk and mortality after solid organ transplantation: A population-based 30-year cohort study in Finland. Int. J. Cancer.

[8] Au E.H., Chapman J.R., Craig J.C., Lim W.H., Teixeira-Pinto A., Ullah S., McDonald S., Wong G. (2019). Overall and Site-Specific Cancer Mortality in Patients on Dialysis and after Kidney Transplant. J. Am. Soc. Nephrol..

[9] Yeh C.-C., Khan A., Muo C.-H., Yang H.-R., Li P.-C., Chang C.-H., Chen T.-L., Jeng L.-B., Liao C.-C. (2020). De Novo Malignancy After Heart, Kidney, and Liver Transplant: A Nationwide Study in Taiwan. Exp. Clin. Transplant..

[10] Krynitz B., Edgren G., Lindelöf B., Baecklund E., Brattström C., Wilczek H., Smedby K.E. (2013). Risk of skin cancer and other malignancies in kidney, liver, heart and lung transplant recipients 1970 to 2008—A Swedish population-based study. Int. J. Cancer.

[11] Collett D., Mumford L., Banner N.R., Neuberger J., Watson C. (2010). Comparison of the incidence of malignancy in recipients of different types of organ: A UK registry audit. Am. J. Transplant..

[12] Kasiske B.L., Snyder J.J., Gilbertson D.T., Wang C. (2004). Cancer after kidney transplantation in the United States. Am. J. Transplant..

[13] Villeneuve P.J., Schaubel D.E., Fenton S.S., Shepherd F.A., Jiang Y., Mao Y. (2007). Cancer incidence among canadian kidney transplant recipients. Am. J. Transplant..

[14] Kitchlu A., Reid J., Jeyakumar N., Dixon S.N., Munoz A.M., Silver S.A., Booth C.M., Chan C.T., Garg A.X., Amir E. (2022). Cancer Risk and Mortality in Patients With Kidney Disease: A Population-Based Cohort Study. Am. J. Kidney Dis..

[15] Blosser C.D., Haber G., Engels E.A. (2021). Changes in cancer incidence and outcomes among kidney transplant recipients in the United States over a thirty-year period. Kidney Int..

[16] Taborelli M., Serraino D., Cimaglia C., Furian L., Biancone L., Busnach G., Todeschini P., Bossini N., Iaria M., Campise M.R. (2022). The impact of cancer on the risk of death with a functioning graft of Italian kidney transplant recipients. Am. J. Transplant..

[17] Taborelli M., Serraino D., Cimaglia C., Furian L., Biancone L., Busnach G., Bossini N., Citterio F., Veroux M., Iaria M. (2024). Cancer mortality after kidney transplantation: A multicenter cohort study in Italy. Int. J. Cancer.

[18] Laowalert S., Naitook N., Boonnim K., Prungrit U., Aekkachaipitak N., Lamjantuek P., Liwlompaisan W., Khunprakant R., Techawathanawanna N., Mavichak V. (2024). Report on post-transplantation cancer in southeast Asia from the Thai kidney transplantation cohort. Sci. Rep..

[19] Cheung C.Y., Lam M.F., Chu K.H., Chow K.M., Tsang K.Y., Yuen S.K., Wong P.N., Chan S.K., Leung K.T., Chan C.K. (2012). Malignancies after kidney transplantation: Hong Kong renal registry. Am. J. Transplant..

[20] Li W.-H., Chen Y.-J., Tseng W.-C., Lin M.-W., Chen T.-J., Chu S.-Y., Hwang C.-Y., Chen C.-C., Lee D.-D., Chang Y.-T. (2012). Malignancies after renal transplantation in Taiwan: A nationwide population-based study. Nephrol. Dial. Transplant..

[21] Tsaur I., Karalis A., Probst M., Blaheta R.A., Scheuermann E., Gossmann J., Kachel H., Hauser I.A., Jonas D., Obermüller N. (2010). Development of urological cancers in renal transplant recipients: 30-year experience at the Frankfurt Transplant Center. Cancer Sci..

[22] Jung S.W., Lee H., Cha J.M. (2022). Risk of malignancy in kidney transplant recipients: A nationwide population-based cohort study. BMC Nephrol..

[23] Oliveras L., Pareja L., Ribes J., Comas J., Couceiro C., Favà À., Codina S., Coloma A., Manonelles A., Lloberas N. (2025). Cancer risks in people on dialysis and kidney transplant recipients: A Catalan cohort study, 2003–2021. Clin. Kidney J..

[24] Ietto G., Gritti M., Pettinato G., Carcano G., Gasperina D.D. (2023). Tumors after kidney transplantation: A population study. World J. Surg. Oncol..

[25] Nimmo A., Elyan B., Lakey J., Marjoribanks S., Methven S., Morrison D., Bell S. (2025). Increased cancer risk in kidney transplant patients in Scotland: A national registry linkage study. Br. J. Cancer.

[26] Srisuwarn P., Sutharattanapong N., Disthabanchong S., Kantachuvesiri S., Kitiyakara C., Phakdeekitcharoen B., Ingsathit A., Sumethkul V. (2024). Incidence of De Novo Post-Transplant Malignancies in Thai Adult Kidney Transplant Recipients: A Single-Center, Population-Controlled, Retrospective Cohort Study at the Highest Volume Kidney Transplant Center in Thailand. Transpl. Int..

[27] Komorowska-Jagielska K., Dębska-Ślizień A., Kolonko A., Heleniak Z., Ruszkowski J., Czarnacka K., Imko-Walczuk B., Biedunkiewicz B., Bułło-Piontecka B., Bzoma B. (2025). Retrospective assessment of the frequency of cancer in the population of kidney transplant recipients—The experience of two transplant centers. Front. Oncol..

[28] Lizakowski S., Kolonko A., Imko-Walczuk B., Komorowska-Jagielska K., Rutkowski B., Więcek A., Dębska-Ślizień A. (2018). Solid Organ Cancer and Melanoma in Kidney Transplant Recipients: TumorTx Base Preliminary Results. Transpl. Proc..

[29] Kalil R.S., Lynch C.F., Engels E.A. (2015). Risk of cancer in retransplants compared to primary kidney transplants in the United States. Clin. Transplant..

[30] Gioco R., Corona D., Agodi A., Privitera F., Barchitta M., Giaquinta A., Alba I., D’eRrico S., Pinto F., De Pasquale C. (2019). De Novo Cancer Incidence and Prognosis After Kidney Transplantation: A Single Center Analysis. Transpl. Proc..

[31] Krishnan A., Wong G., Teixeira-Pinto A., Lim W.H. (2022). Incidence and Outcomes of Early Cancers After Kidney Transplantation. Transpl. Int..

[32] D’aRcy M.E., Coghill A.E., Lynch C.F., Koch L.A., Li J., Pawlish K.S., Morris C.R., Rao C., Engels E.A. (2019). Survival after a cancer diagnosis among solid organ transplant recipients in the United States. Cancer.

[33] Farrugia D., Cheshire J., Begaj I., Khosla S., Ray D., Sharif A. (2014). Death within the first year after kidney transplantation—An observational cohort study. Transpl. Int..

[34] Mazzucotelli V., Piselli P., Verdirosi D., Cimaglia C., Cancarini G., Serraino D., Sandrini S. (2017). De novo cancer in patients on dialysis and after renal transplantation: North-western Italy, 1997–2012. J. Nephrol..

[35] Penn I. (1979). Renal transplantation for wilms tumor: Report of 20 cases. J. Urol..

[36] Penn I. (1995). Primary kidney tumors before and after renal transplantation. Transplantation.

[37] Williams J.C., Merguerian P.A., Schned A.R., Morrison P.M. (1995). Acquired renal cystic disease and renal cell carcinoma in an allograft kidney. J. Urol..

[38] Penn I. (1991). Donor transmitted disease: Cancer. Transplant. Proc..

[39] Nationwide Cancer Database. https://onkologia.org.pl/pl.

[40] Kauffman H.M., McBride M.A., Delmonico F.L. (2000). First report of the United Network for Organ Sharing Transplant Tumor Registry: Donors with a history of cancer. Transplantation.

[41] McHayleh W., Morcos J.P., Wu T., Shapiro R., Yousem S., Appleman L., Friedland D.M. (2008). Renal cell carcinoma from a transplanted allograft: Two case reports and a review of the literature. Clin. Genitourin. Cancer.

[42] Bokemeyer C., Thon W., Brunkhorst T., Kuczyk M., Pichlmayr R., Kliem V. (1996). High frequency of urothelial cancers in patients with kidney transplantations for end-stage analgesic nephropathy. Eur. J. Cancer.

[43] Levine L.A., Gburek B.M. (1994). Acquired cystic disease and renal adenocarcinoma following renal transplantation. J. Urol..

[44] Muruve N.A., Shoskes D.A. (2005). Genitourinary Malignancies in Solid Organ Transplant Recipients. Transplantation.

[45] Pommerolle P., Assem M., Uhl M., De Sousa P., Guerrot D., Hazzan M., Lobbedez T., Fourdinier O., Choukroun G. (2025). Renal Cell Carcinoma in Native Kidney After Kidney Transplantation: A Multicenter Case Control Study With a Focus on Screening Strategy. Transpl. Int..

[46] Bratt O., Drevin L., Prütz K., Carlsson S., Wennberg L., Stattin P. (2020). Prostate cancer in kidney transplant recipients—A nationwide register study. BJU Int..

[47] Konety B.R., Tewari A., Howard R.J., Barry J.M., Hodge E.E., Taylor R., Jordon M.L. (1998). Prostate cancer in the post-transplant population. Urology.

[48] Kleinclauss F., Gigante M., Neuzillet Y., Mouzin M., Terrier N., Salomon L., Iborra F., Petit J., Cormier L., Lechevallier E. (2008). Prostate cancer in renal transplant recipients. Nephrol. Dial. Transplant..

[49] Lin W., Pan X., Zhang C., Ye B., Song J. (2023). Impact of Age at Diagnosis of Bladder Cancer on Survival: A Surveillance, Epidemiology, and End Results-Based Study 2004-2015. Cancer Control.

[50] Zhang Y., Rumgay H., Li M., Yu H., Pan H., Ni J. (2023). The global landscape of bladder cancer incidence and mortality in 2020 and projections to 2040. J. Glob. Health.

[51] Siegel R.L., Kratzer T.B., Giaquinto A.N., Sung H., Jemal A. (2025). Cancer statistics, 2025. CA Cancer J. Clin..

[52] Buzzeo B.D., Heisey D.M., Messing E.M. (1997). Bladder cancer in renal transplant recipients. Urology.

[53] Tuttle T.M., Williams G.M., Marshall F.F. (1988). Evidence for cyclophosphamide-induced transitional cell carcinoma in a renal transplant patient. J. Urol..

[54] Alexiev B.A., Randhawa P., Martul E.V., Zeng G., Luo C., Ramos E., Drachenberg C.B., Papadimitriou J.C. (2013). BK virus–associated urinary bladder carcinoma in transplant recipients: Report of 2 cases, review of the literature, and proposed pathogenetic model. Hum. Pathol..

[55] Bialasiewicz S., Cho Y., Rockett R., Preston J., Wood S., Fleming S., Shepherd B., Barraclough K., Sloots T., Isbel N. (2013). Association of micropapillary urothelial carcinoma of the bladder and BK viruria in kidney transplant recipients. Transpl. Infect. Dis..

[56] Klufah F., Mobaraki G., Shi S., Marcelissen T., Alharbi R.A., Mobarki M., Almalki S.S.R., van Roermund J., Hausen A.Z., Samarska I. (2023). Human polyomaviruses JCPyV and MCPyV in urothelial cell carcinoma: A single institution experience. Front. Oncol..

[57] Basile G., Fallara G., Bandini M., Cazzaniga W., Negri F., Dieguez L., Montorsi F., Salonia A., Breda A., Fankhauser C. (2025). Testis and penile cancers in kidney transplant recipients: A systematic review of epidemiology, treatment options and oncological outcomes by the EAU-YAU Penile and Testis Cancer Working Group. Actas Urol. Esp..

[58] Besarani D., Cranston D. (2007). Urological malignancy after renal transplantation. BJU Int..

[59] Jiang S., Regmi S., Jackson S., Calvert C., Jarosek S., Pruett T., Warlick C. (2020). Risk of Genitourinary Malignancy in the Renal Transplant Patient. Urology.

[60] Piselli P., Serraino D., Segoloni G.P., Sandrini S., Piredda G.B., Scolari M.P., Rigotti P., Busnach G., Messa P., Donati D. (2013). Risk of de novo cancers after transplantation: Results from a cohort of 7217 kidney transplant recipients, Italy 1997–2009. Eur. J. Cancer.

[61] Antunes H., Tavares-Da-Silva E., Oliveira R., Carvalho J., Parada B., Bastos C., Figueiredo A. (2018). De Novo Urologic Malignancies in Renal Transplant Recipients. Transplant. Proc..

[62] Elkentaoui H., Robert G., Pasticier G., Bernhard J.-C., Couzi L., Merville P., Ravaud A., Ballanger P., Ferrière J.-M., Wallerand H. (2010). Therapeutic Management of De Novo Urological Malignancy in Renal Transplant Recipients: The Experience of the French Department of Urology and Kidney Transplantation from Bordeaux. Urology.

[63] Kim H., Chae K.-H., Choi A., Kim M.-H., Hong J.H., Choi B.S., Kim S., Ban T.H. (2025). Increased risk of genitourinary cancer in kidney transplant recipients: A large-scale national cohort study and its clinical implications. Int. Urol. Nephrol..

[64] Minkovich M., Wong R.B.K., Famure O., Li Y., Kim S.J., Lee J.Y. (2023). Renal cell carcinoma in kidney transplant recipients: Incidence, trends, clinical management & outcomes. World J. Urol..

[65] E D’aRcy M., Castenson D., Lynch C.F., Kahn A.R., Morton L.M., Shiels M.S., Pfeiffer R.M., A Engels E. (2021). Risk of Rare Cancers Among Solid Organ Transplant Recipients. JNCI J. Natl. Cancer Inst..

[66] Stakhovskyi O., Yap S.A., Leveridge M., Lawrentchuk N., Jewett M.A.S. (2011). Small renal mass: What the urologist needs to know for treatment planning and assessment of treatment results. Am. J. Roentgenol..

[67] Schwarz A., Vatandaslar S., Merkel S., Haller H. (2007). Renal Cell carcinoma in transplant recipients with acquired cystic kidney disease. Clin. J. Am. Soc. Nephrol..

[68] Reichelt O., Wunderlich H., Neiser G., Schubert J. (2003). How reliable is conventional urinary cytology in post-transplant patients?. Urol. Int..

[69] Klein C., Bosc N., Marty S., Calen L., Debard C., Robert G., Haaser T. (2025). Prostate Cancer Patients’ Perceptions Regarding the Relevance of a Digital Rectal Examination During Their Follow-Up After Radiation Therapy. Cancer Med..

[70] Kirby M., Merriel S.W., Olajide O., Norman A., Vasdev N., Hanchanale V., Cain M., Wilkinson M., Stephens H., Victor D. (2024). Is the digital rectal exam any good as a prostate cancer screening test?. Br. J. Gen. Pr..

[71] Mensah J.E., Akpakli E., Kyei M., Klufio K., Asiedu I., Asante K., Toboh B., Ashaley M.D., Addo B.M., Morton B. (2025). Prostate-specific antigen, digital rectal examination, and prostate cancer detection: A study based on more than 7000 transrectal ultrasound-guided prostate biopsies in Ghana. Transl. Oncol..

[72] Wammack R., Djavan B., Remzi M., Susani M., Marberger M. (2001). Morbidity of transrectal ultrasound-guided prostate needle biopsy in patients receiving immunosuppression. Urology.

[73] Whang M., Sheng J., Chang C., Weiss R.E., Bhalla R., Geffner S., Weng F. (2023). Recommendations for patients with prostate cancer who wish to undergo a kidney transplant. Transplant. Rep..

[74] Master V.A., Meng M.V., Grossfeld G.D., Koppie T.M., Hirose R., Carroll P.R. (2004). Treatment and outcome of invasive bladder cancer in patients after renal transplantation. J. Urol..

[75] Diller R., Gruber A., Wolters H., Senninger N., Spiegel H.-U. (2006). Therapy and prognosis of tumors of the genitourinary tract after kidney transplantation. Transplant. Proc..

[76] Griffith J.J., Amin K.A., Waingankar N., Lerner S.M., Delaney V., Ames S.A., Badani K., Palese M.A., Mehrazin R. (2017). Solid renal masses in transplanted allograft kidneys: A closer look at the epidemiology and management. Am. J. Transplant..

[77] Dahle D.O., Skauby M., Langberg C.W., Brabrand K., Wessel N., Midtvedt K. (2022). Renal Cell Carcinoma and Kidney Transplantation: A Narrative Review. Transplantation.

[78] Krisl J.C., Doan V.P. (2017). Chemotherapy and Transplantation: The Role of Immunosuppression in Malignancy and a Review of Antineoplastic Agents in Solid Organ Transplant Recipients. Am. J. Transplant..

[79] Hanusz K., Domański P., Strojec K., Zapała P., Zapała Ł., Radziszewski P. (2023). Prostate Cancer in Transplant Receivers—A Narrative Review on Oncological Outcomes. Biomedicines.

[80] Grabe-Heyne K., Henne C., Mariappan P., Geiges G., Pöhlmann J., Pollock R.F. (2023). Intermediate and high-risk non-muscle-invasive bladder cancer: An overview of epidemiology, burden, and unmet needs. Front. Oncol..

[81] Chang S.S., Boorjian S.A., Chou R., Clark P.E., Daneshmand S., Konety B.R., Pruthi R., Quale D.Z., Ritch C.R., Seigne J.D. (2016). Diagnosis and treatment of non-muscle invasive bladder cancer: AUA/SUO guideline. J. Urol..

[82] Babjuk M., Burger M., Capoun O., Cohen D., Compérat E.M., Escrig J.L.D., Gontero P., Liedberg F., Masson-Lecomte A., Mostafid A.H. (2022). European association of urology guidelines on non–muscle-invasive bladder cancer (Ta, T1, and Carcinoma in Situ). Eur. Urol..

[83] Matulewicz R.S., Steinberg G.D. (2020). Non-muscle-invasive bladder cancer: Overview and contemporary treatment landscape of neoadjuvant chemoablative therapies. Rev. Urol..

[84] Fong K.Y., Lim E.J., So W.Z., Aslim E.J., Tiong H.Y., Gan V.H.L. (2025). Management of bladder cancer in kidney transplant recipients: A narrative review. Bl. Cancer.

[85] Dell’aTti L., Slyusar V. (2025). De Novo Testicular Cancers Arising After Renal Transplantation: A Narrative Review. Anticancer Res..

[86] Escudier B., Porta C., Schmidinger M., Rioux-Leclercq N., Bex A., Khoo V., Grünwald V., Gillessen S., Horwich A. (2019). on behalf of the ESMO Guidelines Committee. Renal cell carcinoma: ESMO Clinical Practice Guidelines for diagnosis, treatment and follow-up. Ann. Oncol..

[87] Powles T., Albiges L., Bex A., Comperat E., Grünwald V., Kanesvaran R., Kitamura H., McKay R., Porta C., Procopio G. (2024). Renal cell carcinoma: ESMO Clinical Practice Guideline for diagnosis, treatment and follow-up. Ann. Oncol..

[88] Powles T., Bellmunt J., Comperat E., De Santis M., Huddart R., Loriot Y., Necchi A., Valderrama B., Ravaud A., Shariat S. (2024). ESMO Clinical Practice Guideline interim update on first-line therapy in advanced urothelial carcinoma. Ann. Oncol..

[89] Moschini M., Gandaglia G., Dehò F., Salonia A., Briganti A., Montorsi F., on behalf of the ESMO Guidelines Committee (2022). Bladder cancer: ESMO Clinical Practice Guideline for diagnosis, treatment and follow-up. Ann. Oncol..

[90] Fizazi K., Gillessen S., ESMO Guidelines Committee (2023). Updated treatment recommendations for prostate cancer from the ESMO Clinical Practice Guideline considering treatment intensification and use of novel systemic agents. Ann. Oncol..

[91] Parker C., Castro E., Fizazi K., Heidenreich A., Ost P., Procopio G., Tombal B., Gillessen S., ESMO Guidelines Committee (2020). Prostate cancer: ESMO Clinical Practice Guidelines for diagnosis, treatment and follow-up. Ann. Oncol..

[92] Dutcher J.P., Flippot R., Fallah J., Escudier B. (2020). On the shoulders of giants: The evolution of renal cell carcinoma treatment—Cytokines, targeted therapy, and immunotherapy. Am. Soc. Clin. Oncol. Educ. Book.

[93] Motzer R.J., Tannir N.M., McDermott D.F., Aren Frontera O., Melichar B., Choueiri T.K., Plimack E.R., Barthélémy P., Porta C., George S. (2018). Nivolumab plus Ipilimumab versus Sunitinib in Advanced Renal-Cell Carcinoma. N. Engl. J. Med..

[94] Rini B.I., Plimack E.R., Stus V., Gafanov R., Hawkins R., Nosov D., Pouliot F., Alekseev B., Soulières D., Melichar B. (2019). Pembrolizumab plus axitinib versus sunitinib for advanced renal-cell carcinoma. N. Engl. J. Med..

[95] Bedke J., Abu Ghanem Y., Albiges L., Bonn S., Campi R., Capitanio U., Dabestani S., Hora M., Klatte T., Kuusk T. (2025). Updated European Association of Urology Guidelines on the Use of Adjuvant Immune Checkpoint Inhibitors and Subsequent Therapy for Renal Cell Carcinoma. Eur. Urol..

[96] Sandhu G., Adattini J., Gordon E.A., O’Neill N., On behalf of the ADDIKD Guideline Working Group (2022). International Consensus Guideline on Anticancer Drug Dosing in Kidney Dysfunction. https://www.eviq.org.au/clinical-resources/addikd-guideline/4174-anticancer-drug-dosing-in-kidney-dysfunction.

[97] Róg L., Pyrża M., Pihowicz P., Perkowska-Ptasińska A., Małyszko J. (2025). Does a kidney biopsy in a patient undergoing systemic oncological treatment make sense?. Oncol. Clin. Pract..

[98] Claeys E., Vermeire K. (2019). Immunosuppressive drugs in organ transplantation to prevent allograft rejection: Mode of action and side effects. J. Immunol. Sci..

[99] Bolufer M., Soler J., Molina M., Taco O., Vila A., Macía M. (2024). Immunotherapy for Cancer in Kidney Transplant Patients: A Difficult Balance Between Risks and Benefits. Transpl. Int..

[100] Barbir E.B., Abdulmoneim S., Dudek A.Z., Kukla A. (2024). Immune Checkpoint Inhibitor Therapy for Kidney Transplant Recipients—A Review of Potential Complications and Management Strategies. Transpl. Int..

[101] Murakami N., Mulvaney P., Danesh M., Abudayyeh A., Diab A., Abdel-Wahab N., Abdelrahim M., Khairallah P., Shirazian S., Kukla A. (2021). A multi-center study on safety and efficacy of immune checkpoint inhibitors in cancer patients with kidney transplant. Kidney Int..

[102] Cui X., Yan C., Xu Y., Li D., Guo M., Sun L., Zhu Z. (2023). Allograft rejection following immune checkpoint inhibitors in solid organ transplant recipients: A safety analysis from a literature review and a pharmacovigilance system. Cancer Med..

[103] Garg N., Rennke H.G., Pavlakis M., Zandi-Nejad K. (2018). De novo thrombotic microangiopathy after kidney transplantation. Transplant. Rev..

[104] Legris T., Sallée M., Charmetant X., Thaunat O., Matignon M., Joher N., Pernin V., Sicard A., Kaminski H., Couzi L. (2025). Immune Checkpoint Inhibitors in Kidney Transplant Recipients: A French Multicenter Retrospective Cohort Study. Transplant. Direct.

[105] Kumar V., Shinagare A.B., Rennke H.G., Ghai S., Lorch J.H., Ott P.A., Rahma O.E. (2020). The Safety and Efficacy of Checkpoint Inhibitors in Transplant Recipients: A Case Series and Systematic Review of Literature. Oncologist.

[106] Remon J., Auclin E., Zubiri L., Schneider S., Rodriguez-Abreu D., Minatta N., Gautschi O., Aboubakar F., Muñoz-Couselo E., Pierret T. (2024). Immune checkpoint blockers in solid organ transplant recipients and cancer: The Innovated cohort. ESMO Open.

[107] Fisher J., Zeitouni N., Fan W., Samie F.H. (2020). Immune checkpoint inhibitor therapy in solid organ transplant recipients: A patient-centered systematic review. J. Am. Acad. Dermatol..

[108] Saleem N., Wang J., Rejuso A., Teixeira-Pinto A., Stephens J.H., Wilson A., Kieu A., Gately R.P., Boroumand F., Chung E. (2025). Outcomes of Solid Organ Transplant Recipients With Advanced Cancers Receiving Immune Checkpoint Inhibitors: A Systematic Review and Individual Participant Data Meta-Analysis. JAMA Oncol..

[109] Kommer A., Stortz M., Kraus D., Weinmann-Menke J. (2025). Immune Checkpoint Inhibitor-Associated Acute Kidney Injury: A Single-Center Experience of Biopsy-Proven Cases. J. Clin. Med..

[110] Zhou P., Gao Y., Kong Z., Wang J., Si S., Han W., Li J., Lv Z., Wang R. (2024). Immune checkpoint inhibitors and acute kidney injury. Front. Immunol..

[111] Renaghan A.D., Ostermann M., Ronco C., Ballen K., Cosmai L., Fenoglio R., Floris M., Forni L.G., Gladstone D.E., Glezerman I.G. (2025). The nephrotoxic effects of anti-cancer therapies: Consensus report of the 34th Acute Disease Quality Initiative workgroup. Nat. Rev. Nephrol..

[112] Vallianou K., Bellos I., Filiopoulos V., Skalioti C., Lagiou P., Benetou V., Marinaki S. (2025). Risk Factors for the Development of Malignancies Post-Transplantation in Kidney Transplant Recipients. Biomedicines.

[113] Yanik E.L., Siddiqui K., Engels E.A. (2015). Sirolimus effects on cancer incidence after kidney transplantation: A meta-analysis. Cancer Med..

[114] Hellemans R., Pengel L.H., Choquet S., Maggiore U., for ESOT Workstream 3 of the TLJ (Transplant Learning Journey) project (2021). Managing immunosuppressive therapy in potentially cured post-kidney transplant cancer (excluding non-melanoma skin cancer): An overview of the available evidence and guidance for shared decision-making. Transpl. Int..

[115] Schenk K.M., Deutsch J.S., Chandra S., Davar D., Eroglu Z., Khushalani N.I., Luke J.J., Ott P.A., Sosman J.A., Aggarwal V. (2024). Nivolumab + Tacrolimus + Prednisone ± Ipilimumab for Kidney Transplant Recipients With Advanced Cutaneous Cancers. J. Clin. Oncol..

[116] Miao Y., Everly J.J., Gross T.G., Tevar A.D., First M.R., Alloway R.R., Woodle E.S. (2009). De novo cancers arising in organ transplant recipients are associated with adverse outcomes compared with the general population. Transplantation.

[117] (2020). Kidney Disease: Improving Global Outcomes (KDIGO) Kidney Transplant Candidate Work Group. KDIGO Clinical Practice Guideline on the Evaluation and Management of Candidates for Kidney Transplantation. Transplantation.

[118] Małyszko J., Bamias A., Danesh F.R., Dębska-Ślizień A., Gallieni M., Gertz M.A., Kielstein J.T., Tesarova P., Wong G., Cheung M. (2021). KDIGO Controversies Conference on onco-nephrology: Kidney disease in hematological malignancies and the burden of cancer after kidney transplantation. Kidney Int..

[119] Al-Adra D.P., Hammel L., Roberts J., Woodle E.S., Levine D., Mandelbrot D., Verna E., Locke J., D’cUnha J., Farr M. (2021). Pretransplant solid organ malignancy and organ transplant candidacy: A consensus expert opinion statement. Am. J. Transplant..

[120] Al-Adra D.P., Hammel L., Roberts J., Woodle E.S., Levine D., Mandelbrot D., Verna E., Locke J., D’cUnha J., Farr M. (2021). Preexisting melanoma and hematological malignancies, prognosis, and timing to solid organ transplantation: A consensus expert opinion statement. Am. J. Transplant..

[121] Vieira M.B., Arai H., Nicolau C., Murakami N. (2024). Cancer Screening and Cancer Treatment in Kidney Transplant Recipients. Kidney360.

[122] (2009). Kidney Disease: Improving Global Outcomes (KDIGO) Transplant Work Group. KDIGO clinical practice guideline for the care of kidney transplant recipients. Am. J. Transplant..

[123] Kasiske B.L., Vazquez M.A., Harmon W.E., Brown R.S., Danovitch G.M., Gaston R.S., Roth D., Scandling J.D., Singer G.G. (2000). Recommendations for the outpatient surveillance of renal transplant recipients. J. Am. Soc. Nephrol..

[124] Wong G., Howard K., Webster A.C., Chapman J.R., Craig J.C. (2011). Screening for renal cancer in recipients of kidney transplants. Nephrol. Dial. Transplant..

[125] EBPG Expert Group on Renal Transplantation (2002). European best practice guidelines for renal transplantation. Section IV: Long-term management of the transplant recipient. I.V.6.3. Cancer risk after renal transplantation. Solid organ cancers: Prevention and treatment. Nephrol. Dial. Transplant..

[126] Faba O.R., Boissier R., Budde K., Figueiredo A., Taylor C.F., Hevia V., García E.L., Regele H., Zakri R.H., Olsburgh J. (2018). European association of urology guidelines on renal transplantation: Update 2018. Eur. Urol. Focus.

[127] Diana P., Klatte T., Amparore D., Bertolo R., Carbonara U., Erdem S., Ingels A., Kara O., Marandino L., Marchioni M. (2023). Screening programs for renal cell carcinoma: A systematic review by the EAU young academic urologists renal cancer working group. World J. Urol..

[128] Yohannan B., Sridhar A., Kaur H., DeGolovine A., Maithel N. (2023). Screening for renal cell carcinoma in renal transplant recipients: A single-centre retrospective study. BMJ Open.

[129] Scandling J.D. (2007). Acquired cystic kidney disease and renal cell cancer after transplantation: Time to rethink screening?. Clin. J. Am. Soc. Nephrol..

[130] Becher E., Wang A., Lepor H. (2019). Prostate Cancer Screening and Management in Solid Organ Transplant Candidates and Recipients. Clin. J. Am. Soc. Nephrol..

[131] Vitiello G.A., Sayed B.A., Wardenburg M., Perez S.D., Keith C.G., Canter D.J., Ogan K., Pearson T.C., Turgeon N. (2016). Utility of Prostate Cancer Screening in Kidney Transplant Candidates. J. Am. Soc. Nephrol..

[132] Fernandes E.T., Manivel J.C., Reddy P.K., Ercole C.J. (1996). Cyclophosphamide associated bladder cancer—A highly aggressive disease: Analysis of 12 cases. J. Urol..

[133] Holzbeierlein J.M., Bixler B.R., Buckley D.I., Chang S.S., Holmes R., James A.C., Kirkby E., McKiernan J.M., Schuckman A.K. (2024). Diagnosis and treatment of non-muscle invasive bladder cancer: AUA/SUO guideline: 2024 amendment. J. Urol..

[134] Monach P.A., Arnold L.M., Merkel P.A. (2010). Incidence and prevention of bladder toxicity from cyclophosphamide in the treatment of rheumatic diseases: A data-driven review. Arthritis Rheum.

[135] Port F.K., Ragheb N.E., Schwartz A.G., Hawthorne V.M. (1989). Neoplasms in Dialysis Patients: A Population-Based Study. Am. J. Kidney Dis..

[136] Toriu N., Yamamoto S., Matsubara T., Kataoka Y., Sakai K., Funakoshi T., Horimatsu T., Tsukamoto T., Murakami N., Jhaveri K.D. (2024). Cancer diagnosis and prognosis after initiation of hemodialysis: Multicenter Japan CANcer and DialYsis (J-CANDY) study. Clin. Kidney J..

[137] Ceretta M.L., Noordzij M., Luxardo R., De Meester J., Diez J.M.A., Finne P., Heaf J.G., Couchoud C., Kramar R., Collart F. (2018). Changes in co-morbidity pattern in patients starting renal replacement therapy in Europe—Data from the ERA-EDTA Registry. Nephrol. Dial. Transplant..

[138] Róg L., Zawierucha J., Symonides B., Marcinkowski W., Małyszko S.J., Małyszko J. (2025). Malignancy in Dialysis Patients—How Serious Is the Problem, Especially in Relation to Waiting List Status?. Cancers.

[139] Unterrainer C., Opelz G., Döhler B., Süsal C., Collaborative Transplant Study (2019). Pretransplant Cancer in Kidney Recipients in Relation to Recurrent and De Novo Cancer Incidence Posttransplantation and Implications for Graft and Patient Survival. Transplantation.

[140] Acuna S.A., Huang J.W., Daly C., Shah P.S., Kim S.J., Baxter N.N. (2017). Outcomes of Solid Organ Transplant Recipients With Preexisting Malignancies in Remission: A Systematic Review and Meta-Analysis. Transplantation.

[141] Rosales B.M., De La Mata N., Vajdic C.M., Kelly P.J., Wyburn K., Webster A.C. (2020). Cancer Mortality in Kidney Transplant Recipients: An Australian and New Zealand Population-Based Cohort Study, 1980–2013. Int. J. Cancer.

[142] Benoni H., Eloranta S., Dahle D.O., Svensson M.H., Nordin A., Carstens J., Mjøen G., Helanterä I., Hellström V., Enblad G. (2020). Relative and Absolute Cancer Risks Among Nordic Kidney Transplant Recipients—A Population-Based Study. Transpl. Int..

[143] Penn I. (1993). The effect of immunosuppression on pre-existing cancers. Transplantation.

[144] Barrett W.L., First M.R., Aron B.S., Penn I. (1993). Clinical course of malignancies in renal transplant recipients. Cancer.

[145] Serkies K., Dębska-Ślizień A., Kowalczyk A., Lizakowski S., Małyszko J. (2023). Malignancies in adult kidney transplant candidates and recipients: Current status. Nephrol. Dial. Transplant..

[146] Abramowicz D., Cochat P., Claas F.H., Heemann U., Pascual J., Dudley C., Harden P., Hourmant M., Maggiore U., Salvadori M. (2015). European Renal Best Practice Guideline on kidney donor and recipient evaluation and perioperative care. Nephrol. Dial. Transplant..

[147] Piana A., López-Abad A., Lanzillotta B., Pecoraro A., Prudhomme T., Haberal H.B., Di Dio M., Marco B.B., Dönmez M.I., Breda A. (2025). Systematic Review on Upper Urinary Tract Carcinoma in Kidney Transplant Recipients. J. Clin. Med..

